# Extraction and spatio-temporal analysis of phenological dates of winter wheat in north Henan Province of China from 2003 to 2018 based on MODIS NDVI time series

**DOI:** 10.1371/journal.pone.0300486

**Published:** 2024-04-16

**Authors:** Zhifang Gao

**Affiliations:** School of Surveying and Land Information Engineering, Henan Polytechnic University, Jiaozuo, China; Universidade Federal de Uberlandia, BRAZIL

## Abstract

Monitoring of spatio-temporal changes in crop phenology is an important part of the remote sensing of agricultural ecosystems. In this study, the segment turning point method was utilised to determine several phenological dates of winter wheat in northern Henan from 2003 to 2018. The spatio-temporal variation characteristics of these main phenological dates were analyzed, and the effects of temperature and precipitation on phenological changes were investigated. The results showed that: (1) The segment turning point method had strong space-time adaptability, and the RMSE of extracted phenoloical dates of multi-stations in a single year or single station in multi-years was less than 10d. (2) Roughly bounded by 114°E, the trefoil stage, tillering stage, overwintering stage and rising stage of winter wheat in the west were earlier than those in the east of northern Henan in 2018. (3) From 2003 to 2018, the interannual change rates of the trefoil date, tillering date, overwintering date, rising date, booting date, and milky date of winter wheat were 6.92 d/10a, 4.36 d/10a, 0.74 d/10a, -0.1 d/10a, -3.97 d/10a and -2.91 d/10a, indicating the trend of delaying pre-winter phenology and advancing post-winter phenology. (4) The delay of pre-winter phenology and the advance of post-winter phenology of winter wheat were significantly related to the increase in growing season temperature. The results of the study should provide a basis for further understanding of the effects of climate change on winter wheat phenology and to provide a reference for remote sensing monitoring of winter wheat phenology.

## 1. Introduction

Crop phenology is a periodic index of the crop growth process, which plays an essential role as an indicator of changes in temperature, precipitation, and other environmental factors and is an important reference for field management. Winter wheat is one of the most important food crops. In the past few decades, global warming has advanced the flowering and ripening stages of winter wheat, shortened its growing period, and significantly affected winter wheat yield [1–3]. Moreover, accurate determination and spatiotemporal variation of winter wheat phenology will help to understand its response to climate change. It is significant to winter wheat field management, yield prediction, and food security.

The traditional method of visual field observation is limited in time and space, cannot realise large-scale synchronous monitoring of crop phenology and is susceptible to human factors. Remote sensing technology can make up for the shortcomings of traditional methods [4], where moderate-spatial resolution optical remote sensing data with the high temporal resolution have absolute advantages in large-scale crop phenology remote sensing extraction [5]. In many works of literature, different filters and curve fitting methods were selected to reconstruct the time series of vegetation indices (VI) [6–13]. Additionally, remote sensing monitoring and spatio-temporal variation investigating of vegetation phenology in different regions were studied by combining the threshold method [6–9], the maximum slope method [10], the turning point method and others [11–13]. The methods of time series reconstruction include best index slope extraction (BISE), the temporal windows operation (TWO), the mean-value iteration filter (MVI), the Savitzky-Golay filter (SG), harmonic analysis of time series (HANTS), asymmetric Gaussian function-fitting, double logistic function-fitting, etc [14]. Among them, the SG filter can retain local mutation information in vegetation growth, which has been widely used [15–20], but it often causes overfitting and sawtooth phenomenon [21]. In contrast, the curve fitting method can reconstruct a smooth VI time series curve, but is affected by strong noise that causes curve offsets. In terms of phenological stage extraction, the threshold method (including the dynamic threshold method) sets reference values for observational indicators as thresholds for the beginning and end of the vegetation growing season [6, 7]. It is simple and easy to use. However, it is sensitive to noise and lacks long-term stability, and the threshold chosen has no potential biophysical significance. The maximum slope method, also known as the derivative method [10], refers to calculating the rate of change of the NDVI time profile and taking the date corresponding to the maximum or minimum value of the slope as the start time of the corresponding phenological period. The turning point method [4] also uses the slope curve to identify the phenological date. This method detects the moment of transition between two states on the NDVI time profile. The physical meaning of the maximum slope method and the turning point method is clearer, but there is a high degree of uncertainty in the correspondence between turning points and ground-observed phenology due to regional and growth differences. Huang et al. [22] used vegetation index time series data combined with historical accumulated temperature and crop model to extract the phenological periods of winter wheat and achieved good results, but such methods have high data requirements and are relatively complex to apply. So far, no existing method or combination has shown consistent performance across regions [23].

In addition, the phenological period of winter wheat in agricultural phenology can be divided into the sowing stage, seedling stage, trefoil stage, tillering stage, overwintering stage, regreening stage, rising stage, jointing stage, booting stage, heading stage, flowering stage, milky stage, maturity stage and so on [15]. Meanwhile, compared with other vegetation, there are two peaks of vegetation index before and after wintering in the growth process of winter wheat. Current remote sensing studies of winter wheat phenology are most concerned with the regreening, heading, and maturity stages [15–17, 20], with less attention paid to pre-winter phenology [18]. However, the growth process of winter wheat before overwintering is a critical growth stage for winter wheat, which accumulates nutrients for later growth and has an essential impact on the growth trend of the overwintering stage and even on the final yield of winter wheat. Moreover, effectively extracting the pre-winter phenology of winter wheat needs further study. At the same time, the VI changes observed by remote sensing could not directly reflect the physiological process of vegetation, and the monitored vegetation dynamics were generally referred to as “land surface phenology” (LSP) [14]. In summary, the research on the correspondence between winter wheat LSP and ground observation phenology remained the focus of current phenological remote sensing research.

Henan Province is the leading province of winter wheat production in China. The wheat production in northern Henan Province holds a significant position in terms of both quality and yield. Current research on pre-winter phenology of winter wheat in northern Henan Province is limited, and the interpretation of the corresponding relationship between LSP and ground-observed phenology varies. In order to clarify the corresponding relationship between LSP and agricultural phenological periods of winter wheat in northern Henan Province, a "segment turning point method" was used in this study. This method extracts absolute value extreme points of NDVI slope by segments, based on the characteristics of NDVI time series slope curve of winter wheat. The start dates of key phenological periods of winter wheat in northern Henan Province from 2003 to 2018 were extracted. This involves the date of pre-winter trefoil and tillering, enabling a more comprehensive representation of phenological changes. At the same time, the spatio-temporal variation characteristics of the key phenological dates of winter wheat in northern Henan from 2003 to 2018 were analyzed. The effects of temperature and precipitation on the phenological changes were preliminarily discussed to provide a more comprehensive reference for the remote sensing monitoring of winter wheat phenological periods and the research of climate change response in the study area.

## 2. Research area and data

### 2.1 Overview

**Fig 1:**
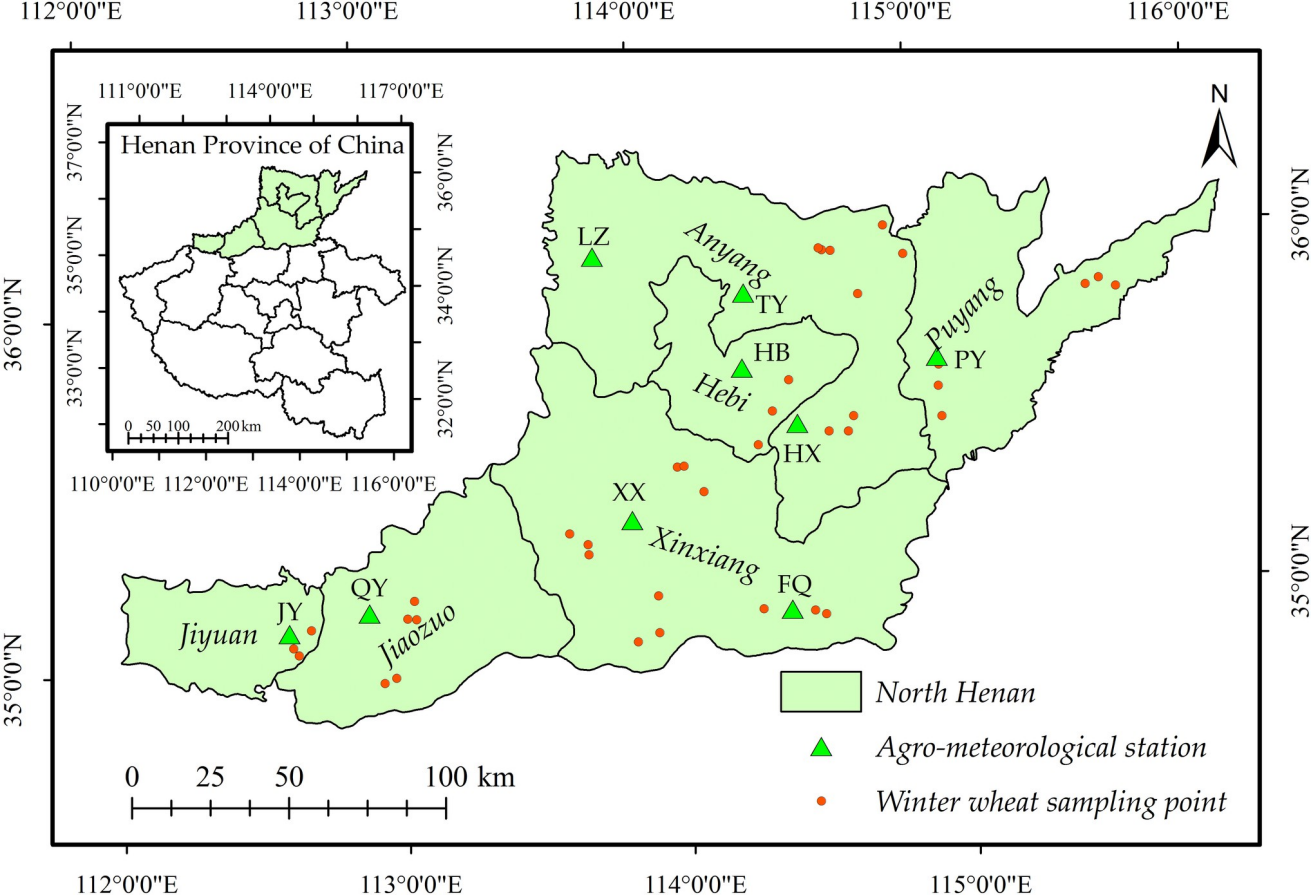
Location and distribution of winter wheat samples and ago-meteorological stations in North Henan Province. The ago-meteorological station numbers LZ, TY, PY, HB, HX, XX, QY, JY and FQ correspond to Linzhou, Tangyin, Puyang, Hebi, Huaxian, Xinxiang, Qinyang, Jiyuan and Fengqiu stations respectively. The administrative boundary data comes from Resource and Environment Science and Data Center (https://www.resdc.cn/).

North Henan is located in the southern Taihang Piedmont Plain, north of the Yellow River in Henan Province, including 6 cities (Anyang, Xinxiang, Jiaozuo, Puyang, Hebi and Jiyuan, Fig 1). The terrain is high in the west and low in the east, covering middle mountains, low mountains, hills, intermountain basins, piedmont alluvial plains and riverine plains. The climate is a warm temperate continental monsoon climate with four distinct seasonal characteristics and the rain and heat corresponding period. The area’s average annual temperature from 1995 to 2018 was 14.7°C, with an average annual precipitation of 569 mm and 1994 annual sunshine hours. There are differences between the east and west regions, with the eastern region having an average annual temperature of 14.4°C, 571 mm of precipitation, and 2014 annual sunshine hours, while the western region has an average annual temperature of 15.1°C, 567 mm of precipitation, and 1970 annual sunshine hours. The main soil types are brown soil and yellow tide soil. The main local crops are winter wheat, corn, cotton, peanut, rapeseed, vegetables, Chinese yam and other medicinal materials. It is also an important national high-quality wheat production base of China. The direction of the Taihang Mountains in the region changes from east-west to north-south. Due to the barrier effect of the Taihang Mountains, the growth conditions of winter wheat in the west and east of northern Henan vary greatly, with distinct regional characteristics. This area is selected as the research area because it is representative to study the response mechanism of the winter wheat growth process to local microclimate under the longitude and latitude. The main sowing period of winter wheat in the study area is the first and middle of October, and the tillering begins in the middle of November. Next, the first and middle of March of the following year is the regreening stage. Then, the heading stage is middle and late April. After, the maturity begins in the middle and late May. Finally, the harvest stage is from the end of May to the first and middle of June.

### 2.2 Data and preprocessing

#### 2.2.1 MODIS NDVI data and processing

Moderate-resolution imaging spectrometer (MODIS) is the main sensor on Terra and Aqua satellites. It can complete a global observation in 1-2d. The MOD13Q1 and MYD13Q1 data used in this study were MODIS Terrestrial Level 3 standard data products with a spatial resolution of 250m and a temporal resolution of 16d, combined into an 8d temporal resolution, in sinusoidal projection, with the tile number H27V05, available from the NASA website (https://ladsweb.modaps.eosdis.nasa.gov/). According to the phenological calendar of winter wheat in northern Henan, the MODIS NDVI data of 33 issues covering the pre-sowing stage to the mature stage of winter wheat from the 273rd day of the year (September 29 or September 30) to the 161st day of the next year (June 9 or June 10) was selected as an NDVI time sequence of winter wheat growth period, forming the MODIS NDVI time series of winter wheat growth periods for a total of 16 years from 2003 to 2018 (from 2002273 to 2003161, recorded as 2003). Meanwhile, the MODIS data were converted into the UTM-WGS84 projection coordinate system in batches using the re-projection tool MRT (MODIS Projection Tool). Then, it was clipped with the boundary vector map. The administrative boundary data comes from Resource and Environment Science and Data Center (https://www.resdc.cn/DOI/doi.aspx?DOIid=121) [24].

#### 2.2.2 Accuracy verification data

The sample data of the winter wheat field survey, statistical data of planting area and phenological observation data of agro-meteorological stations were used for accuracy verification. From March 2017 to March 2018, field surveys were carried out twice in six cities in northern Henan, and 114 winter wheat samples were collected in 37 villages (Fig 1) to verify the accuracy of winter wheat extraction in 2018. The extraction accuracy of the winter wheat in other years was verified by statistical data. The statistical data on winter wheat planting area were collected from the Statistical Yearbook of Henan Province in 2003–2019 (https://www.henan.gov.cn/zwgk/zfxxgk/fdzdgknr/tjxx/tjnj/). The accuracy of the phenological date extraction was verified using agro-meteorological station data from the China Meteorological Data Network (http://data.cma.cn/). In this paper, the data from 9 agro-meteorological stations in northern Henan during the study period (Fig 1) were used, including the titles and start times of the main crop development periods.

In addition, this paper also used the monthly temperature and precipitation data from 27 meteorological stations in northern Henan from 2003 to 2018 to analyze the correlation between the temporal and spatial changes of winter wheat phenological dates and meteorological factors.

## 3. Research method

### 3.1 MODIS NDVI time series reconstruction

Due to the atmospheric and surface bidirectional reflection, the original MODIS NDVI time series data contained a lot of noise and null values. Therefore, it is necessary to reconstruct the time series data of the remote sensing vegetation index to remove or reduce the noise level of the data to the maximum extent and improve the data quality, reflecting vegetation dynamics more realistically.

**Fig 2:**
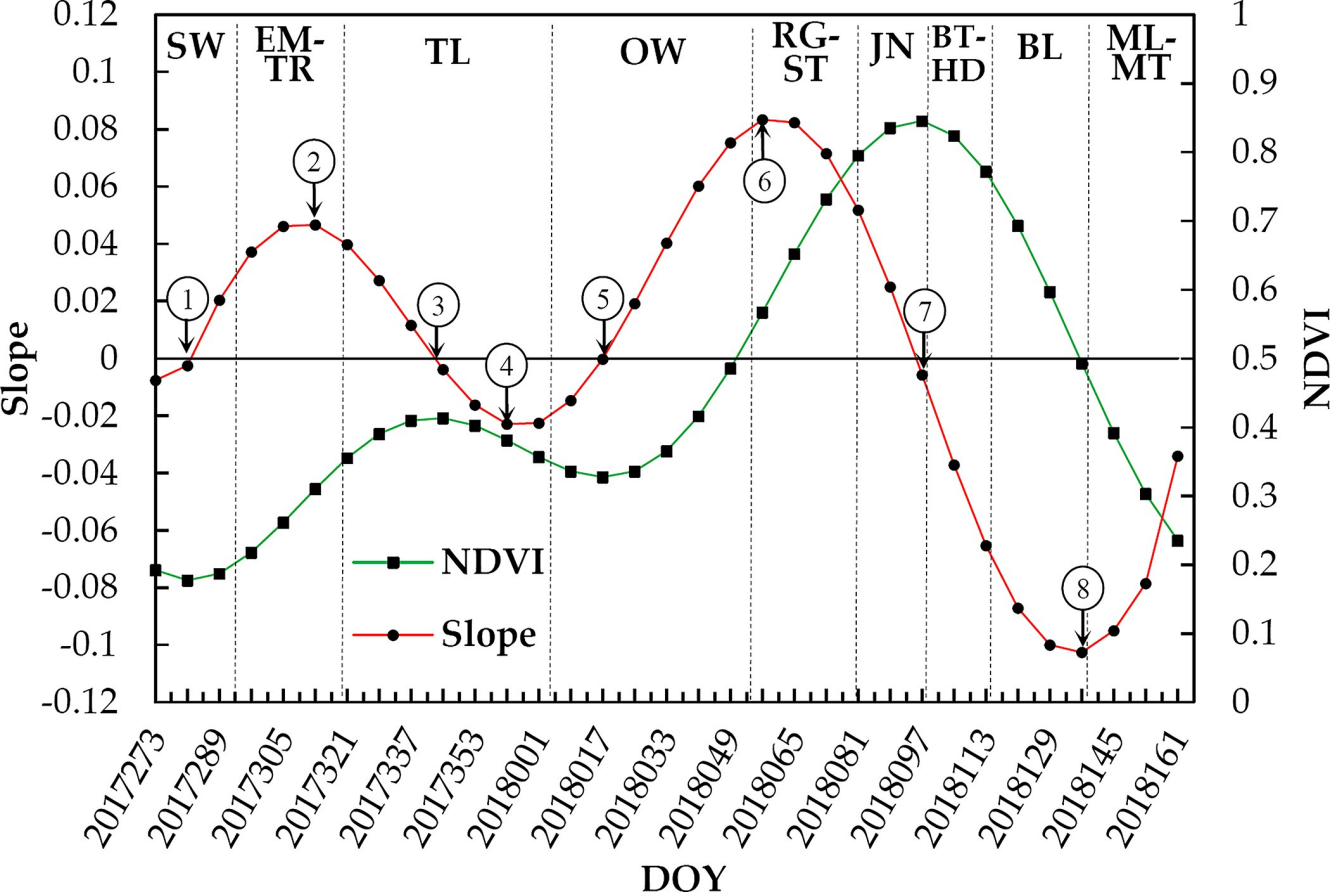
NDVI curve and slope curve of winter wheat growing season near the Xinxiang agro-meteorological station. Where SW for Sowing, EM-TR for Emergence-trefoil, TL for Tillering, OW for Overwintering, RG-RS for Regreening-rising, JN for Jointing, BT-HD for Booting-heading, BL for Blooming and ML-MT for Milky-Mature. The NDVI curve was smoothed by HANTS filter.

HANTS algorithm is a harmonic analysis combining Fourier Transform and least-squares fitting [25]. This method does not need to remove clouds, and the filtered curve is smooth. Meanwhile, the cosine wave has a strong ability to represent the periodic changes of vegetation index, which has certain advantages in phenology extraction and reducing the complexity of classification using time series data [26]. This paper used the HANTS algorithm to reconstruct the NDVI time series data. The reconstructed NDVI curve was smooth with a complete "two peaks and one valley" feature of winter wheat, which was conducive to identifying winter wheat and extracting phenology (Fig 2).

### 3.2 Extraction of winter wheat area

Based on the unique "two peaks and one valley" characteristics of the winter wheat NDVI curve, the decision rule was established by selecting the two peaks before and after winter and the minimum value of the overwintering stage. The winter wheat planting areas in northern Henan from 2003 to 2018 were extracted using the decision tree method. The 114 field samples and the statistical data were used to verify the extraction accuracy of winter wheat areas in 2018 and in other years, respectively. Several metrics were chosen to measure extraction accuracy including producer accuracy, user accuracy and area accuracy. The category’s producer accuracy indicates the correct classification of its reference pixels, while its user accuracy indicates the extent to which other categories are less misclassified [27]. The continuous multi-year winter wheat planting areas from 2003 to 2018 were obtained through the superposition analysis.

### 3.3 Extraction and accuracy verification of winter wheat key phenological dates

Throughout the whole growth period of winter wheat, the NDVI curves had two peaks before and after winter. Meanwhile, the NDVI curve and the NDVI slope value curve each have 4 inflection points, representing the positions of peak and valley values in the curves (Fig 2). These 8 inflection points correspond to the occurrence of the maximum and minimum absolute value of NDVI slope in specific periods of winter wheat (referred to as turning points), following a pattern of alternating increase and decrease. According to the actual observation of winter wheat phenology at the Xinxiang agro-meteorological station in northern Henan, the 8 turning points appeared successively in the sowing stage, the emergence-trefoil stage, the tillering stage, the beginning of the overwintering stage, the overwintering stage, the regreening-rising stage, the jointing-booting-heading stage and the milky-maturity stage.

According to the characteristics of the NDVI growth curve, this paper used a segment turning point method to extract the segmented extreme absolute value of the NDVI slope curve. This method could be comprehensive and convenient to extract the start times of the phenological stages covering the pre- and post- winter growing season of winter wheat. The specific steps are:

1. The original NDVI timing is filtered and smoothed through the HANTS method to eliminate noise interference.
2. The slope of the smoothed NDVI curve is calculated as follows:
$$Slope(t)=\frac{{VI}_{\left(t+1\right)}-{VI}_{\left(t-1\right)}}{2}$$ (1)
  In this formula, *Slope*(*t*) is the slope at time *t*, *VI*_(*t+1*)_ and *VI*_(*t-1*)_ are the NDVI values before and after time *t* respectively.
3. Extract the turning points where *Slope*(*t*) is equal to “0”, then the NDVI slope curve can be divided into 4 segments.
4. Extract the turning points with maximum values of the absolute value of the slope in different segments, where the NDVI changes fastest in the segment.

The turning-points dates correspond to the start dates of the correlated phenological stages.

The corresponding relationships between the dates of each turning point and the specific phenological dates were determined by the principle of minimum mean absolute error (MAE), referring to the phenological observation data of the Xinxiang agro-meteorological station in 2017–2018 and the corresponding turning point dates:

1. The first turning point is the position of the minimum NDVI value before winter. During this period, the bare soil is exposed after the autumn harvest and the MAE was the lowest compared to the observed sowing date. As the remote sensing technology cannot directly observe the sowing situation, the corresponding time of this point was defined as the pre-sowing date (the preparation stage from the land consolidation to the pre-sowing). The occurrence time of this turning point was closely related to the harvest time of the last crop. In northern Henan, winter wheat was generally sown within 1 to 2 weeks after the land consolidation.
2. At the second turning point, which has the smallest MAE with the trilobal date, the third leaf of winter wheat emerges from the second leaf sheath, and the NDVI changes rapidly. The process for winter wheat takes about one week from sowing to emergence, the same time as from emergence to trefoil stage. This point can be used to calculate the sowing and emergence times of winter wheat.
3. The third turning point is the maximum NDVI of the wheat field before winter, which occurs at the tillering stage. However, the tillering stage of winter wheat is more than 3 months. In general, the proportion of winter wheat tillering in northern Henan can reach about 70% before overwintering. The tillering date recorded by the agro-meteorological station is the date when the tip of the first tiller is about 0.5–1.0 cm in the leaf sheath of more than 50% of the plants in the whole field [28]. At this time, the NDVI value of the wheat field has not yet reached its pre-winter maximum. Therefore, the tillering date can be calculated by subtracting the MAE from the turning point date.
4. The fourth turning point corresponds to the time when the NDVI of winter wheat in the overwintering stage declines most rapidly, which is closest to the start time of the overwintering stage.
5. The fifth turning point is the occurrence time of the minimum NDVI value in the overwintering stage, which is in the middle of the overwintering stage, i.e. the dormant period of winter wheat. The threshold method could be used to calculate the rejuvenation period in combination with the maximum value after winter. According to the calculation, the dynamic threshold value in the north of Henan was 0.3.
6. The sixth turning point is when the NDVI increases most rapidly after winter, and has the smallest MAE with the observed rising date. During the rising stage, the winter wheat seedlings change from creeping to upright growth [28].
7. The seventh turning point is where the maximum value of the winter wheat NDVI growth curve occurs and has the minimum MAE value with the observed booting date. At the booting stage, all the flag leaves of winter wheat have emerged from the sheath. Meanwhile, all the leaves of winter wheat are expanded, and the NDVI peaked [29–31]. The spectral information of the winter wheat canopy changes after heading, and the NDVI decreases [20].
8. The 8th turning point is when the NDVI decreases most rapidly after the peak, corresponding to the milky date. After the winter wheat entered the milky stage, the ear and the upper part of the stem begin to turn yellow, and the NDVI decreases rapidly.

The extraction accuracy of the phenological period was verified by calculating the MAE and the root mean square error (RMSE) between the observed and extracted values. 5 winter wheat samples were selected near each of the 9 agro-meteorological stations in northern Henan, and the modes of the extracted phenological dates were taken as the extracted values of the stations in the present study. The smaller the MAE and RMSE, the higher the accuracy.

### 3.4 Analysis of the time change trend of key phenological dates of winter wheat

In this study, pixel-scale univariate linear regression analysis was used to measure the multi-year change trend of the phenological dates at each winter wheat pixel. The univariate linear regression slope of the phenological extraction time and year was calculated pixel by pixel in the continuous multi-year winter wheat planting area. Regression results were tested by F-test at the 0.05 level for significance. If the slope is greater than 0, it means that the phenological date is delayed. Conversely, if the slope is less than 0, it means that the phenological date is advanced. The temporal variation trend of the phenological dates in each region was represented by the regional mean of the regression slope.

## 4. Result and analysis

### 4.1 Extraction results of winter wheat area

**Fig 3:**
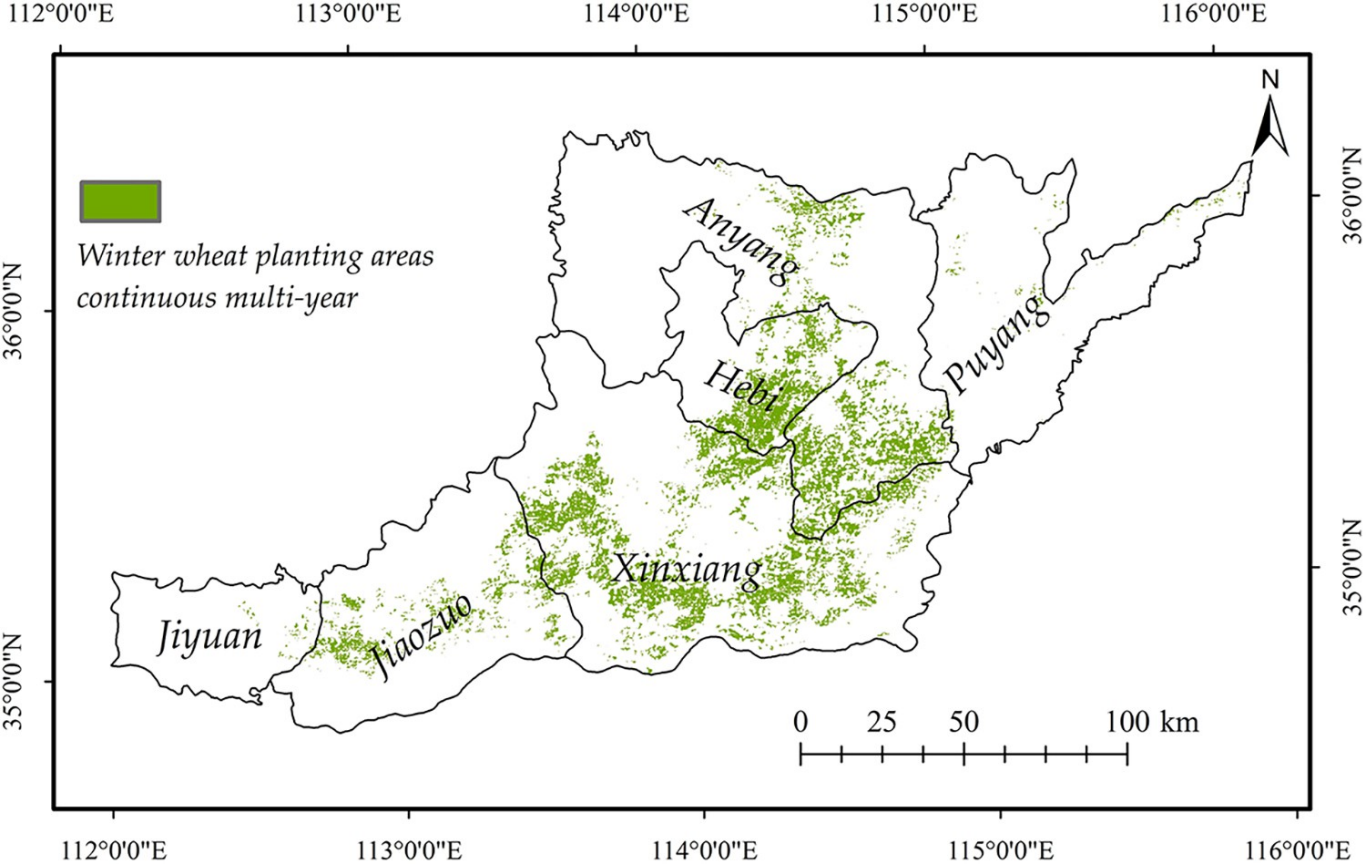
Continuous multi-year winter wheat planting areas in North Henan Province from 2003 to 2018. The administrative boundary data comes from Resource and Environment Science and Data Center (https://www.resdc.cn/).

The decision tree method was used to extract the winter wheat planting areas in northern Henan from 2003 to 2018 yearly (Table 1). Field samples were used to test the accuracy of the confusion matrix for the extraction results of winter wheat planting areas in 2018. The results showed that the producer accuracy and user accuracy of winter wheat planting areas extraction in 2018 were 92.24% and 93.86%, and the Kappa coefficient reached 0.92, with high extraction precision. According to the test of statistical data, the accuracy of the average area extracted from the winter wheat planting area in North Henan from 2003 to 2018 reached 94.36%, with a high correlation between the extracted value and the statistical value (R = 0.92, P<0.01). The extraction results of winter wheat planting areas from 2003 to 2018 were superimposed and analyzed to obtain the continuous multi-year winter wheat planting areas in northern Henan from 2003 to 2018 (Fig 3).

**Table 1:** Accuracy of extracted winter wheat planting areas from 2003 to 2018.

Year	2003	2004	2005	2006	2007	2008	2009	2010
Actual area /kha	978.72	989.49	1017.62	1048.98	1077.67	1087.39	1097.81	1101.34
Extracted area / kha	900.58	953.24	939.36	1012.44	1010.96	993.9	1033.93	1029.54
Area accuracy /%	92.02	96.34	92.31	96.52	93.81	91.4	94.18	93.48
**Year**	**2011**	**2012**	**2013**	**2014**	**2015**	**2016**	**2017**	**2018**
Actual area / kha	1104.11	1109.47	1115.9	1121.97	1125.62	1146.11	1185.89	1209.65
Extracted area / kha	1060.88	1061.33	1042.99	1100.38	1016.14	1120.82	1124.16	1120.83
Area accuracy /%	96.08	95.66	93.47	98.08	90.27	98.67	94.8	92.66

### 4.2 Extraction results of winter wheat phenological dates

The start dates of 7 key phenological periods of winter wheat in the northern Henan Province from 2003 to 2018 were extracted, such as the pre-sowing, trefoil, tillering, overwintering, rising, booting and milky stages (Table 2).

**Table 2:** Extracted results (DOY) and error statistics of key phenological dates of winter wheat in Northern Henan in 2018. The observed pre-sowing dates in the table were taken from the sowing dates recorded at the agro-meteorological stations.

Phenological stage	Phenological date	PY	HX	LZ	TY	HB	XX	FQ	JY	QY	MAE (d)	RMSE (d)
Pre-sowing	Observed	292	287	279	291	288	289	288	297	300	10.4	11.4
	Extracted	281	273	281	273	281	281	273	289	289		
Trefoil	Observed	314	307	303	310	306	306	306	314	320	4.6	6.6
	Extracted	313	313	313	305	305	305	305	313	305		
Tillering	Observed	328	318	314	318	312	322	322	334	338	17	19.3
	Extracted	345	345	329	345	345	337	345	337	337		
Overwintering	Observed	7	332	350	348	4	6	4	4	5	12.2	16.9
	Extracted	1	9	1	1	1	1	1	361	361		
Rising	Observed	81	67	65	65	63	61	71	69	63	4.4	6.4
	Extracted	65	65	65	65	65	65	65	65	57		
Booting	Observed	116	102	106	108	102	98	100	104	100	6.1	7.2
	Extracted	105	97	97	105	89	97	97	97	97		
Milky	Observed	142	136	142	142	138	138	140	142	138	3.9	4.6
	Extracted	137	137	137	137	129	137	137	137	137		
Total error											8.4	11.6

The observed pre-sowing dates in the table were taken from the sowing dates recorded at the agro-meteorological stations.

The total MAE and RMSE of the extracted phenological dates in 2018 were 8.4 d and 11.6 d. Except for the tillering and overwintering dates, the errors of other key phenological dates and the overall error were less than 12 d (1.5 times the time interval), indicating high overall extraction accuracy. The tillering date had the highest error. The error analysis of the observed and extracted dates of winter wheat phenological periods of 16 years from 2003 to 2018 at Xinxiang Station in the central part of northern Henan Province (Table 3) was conducted to verify the extraction effect of the method in this paper on the phenological periods of different years. The total MAE and RMSE of extracted dates of the 7 phenological periods of the Xinxiang station from 2003 to 2018 were 9.3 d and 12.2 d, which were close to those of the 9 agro-meteorological stations in 2018. The results showed that the segment turning point method had high accuracy and strong spatio-temporal adaptability.

**Table 3:** Extraction error statistics of the winter wheat key phenological dates of Xinxiang Station from 2003 to 2018.

	Pre-sowing	Trefoil	Tillering	Overwintering	Rising	Booting	Milky	Total error
MAE(d)	3.9	6.6	20.2	13.2	6.4	8.1	6.9	9.3
RMSE(d)	5.5	8.2	21.5	16.5	7.4	9.0	8.3	12.2

### 4.3 Spatial characteristics of the phenological dates of the winter wheat in northern Henan in 2018

**Fig 4:**
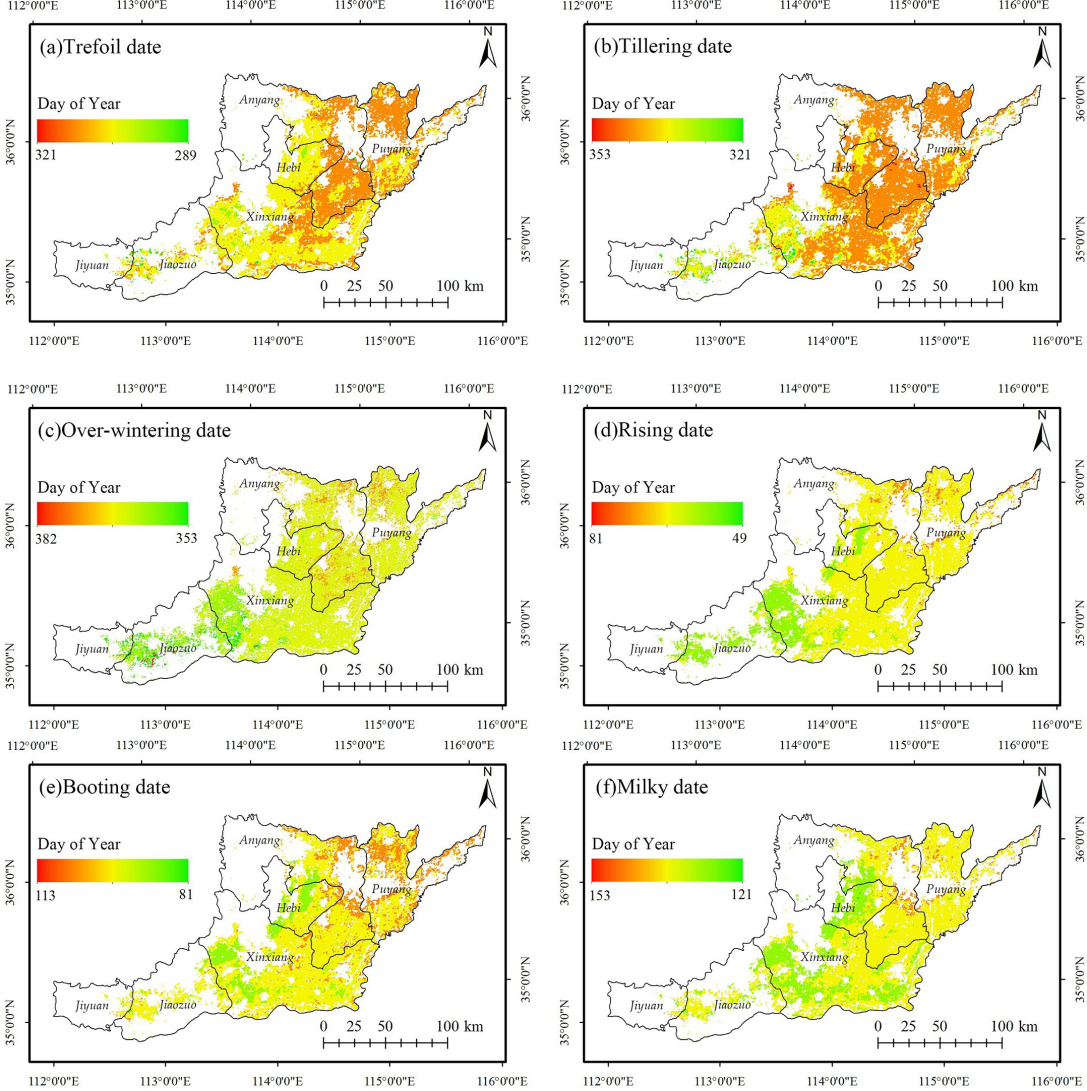
Extracted results of the winter wheat key phenological dates in Northern Henan of 2018. The administrative boundary data comes from Resource and Environment Science and Data Center (https://www.resdc.cn/).

In 2018, the spatial distribution of each phenological date in northern Henan showed obvious spatial differences (Fig 4). In the trefoil stage, the western regions of northern Henan, including Jiyuan, Jiaozuo, Hebi, and most of Xinxiang, were earlier. The trefoil date was relatively late in the strip area composed of western Puyang, eastern Anyang, and eastern Xinxiang (east of Weihe River in Anyang City, west of Jindi River in Puyang City, north of Wenyan Canal in Xinxiang City). The difference between the eastern and western parts of the tillering, overwintering, and Rising stages was obvious. The boundary was about 114°E. The western part of the boundary includes Jiyuan, Jiaozuo, and Xinxiang, which was earlier than the eastern part. At the booting stage, the west of Hebi and Xinxiang, the edge of Taihang Mountain, were the earliest, and the north of Anyang and Puyang was the latest. Other regions are relatively consistent. The milky stage was earlier in the northeast and southeast parts of the Taihang Mountains and Piedmont Plain in northern Henan, along the Yellow River, and later in the scattered areas in northern Anyang. Since the pre-sowing stage is more closely related to the harvest time of the last crop, it will not be discussed in the follow-up analysis.

### 4.4 The changing trend of phenological stages in northern Henan from 2003 to 2018

#### 4.4.1 The changing trend of phenologocal stages at pixel scale in northern Henan

**Fig 5:**
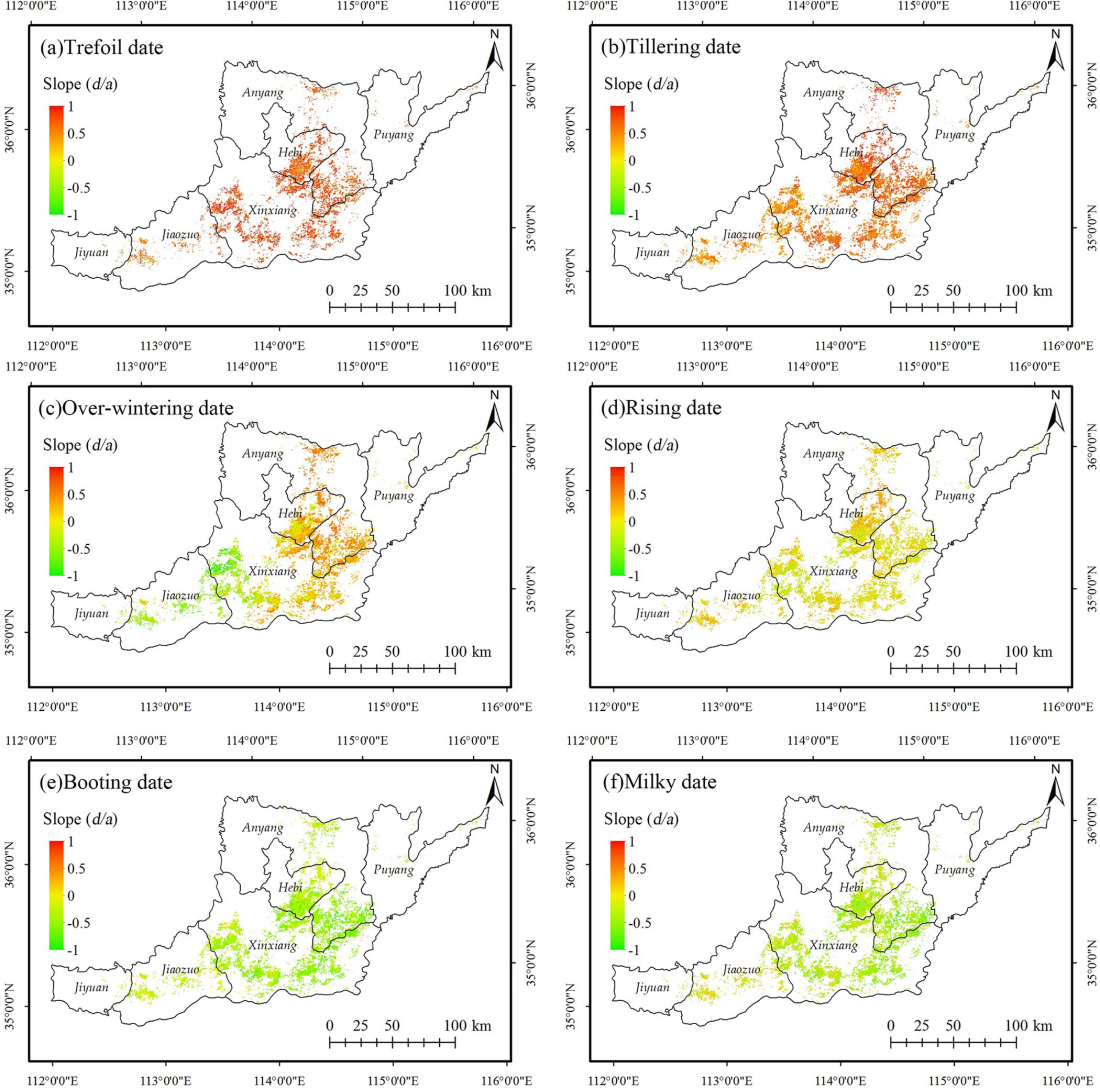
Trends of the key phenological dates of the winter wheat in Northern Henan from 2003 to 2018. The administrative boundary data comes from Resource and Environment Science and Data Center (https://www.resdc.cn/).

**Fig 6:**
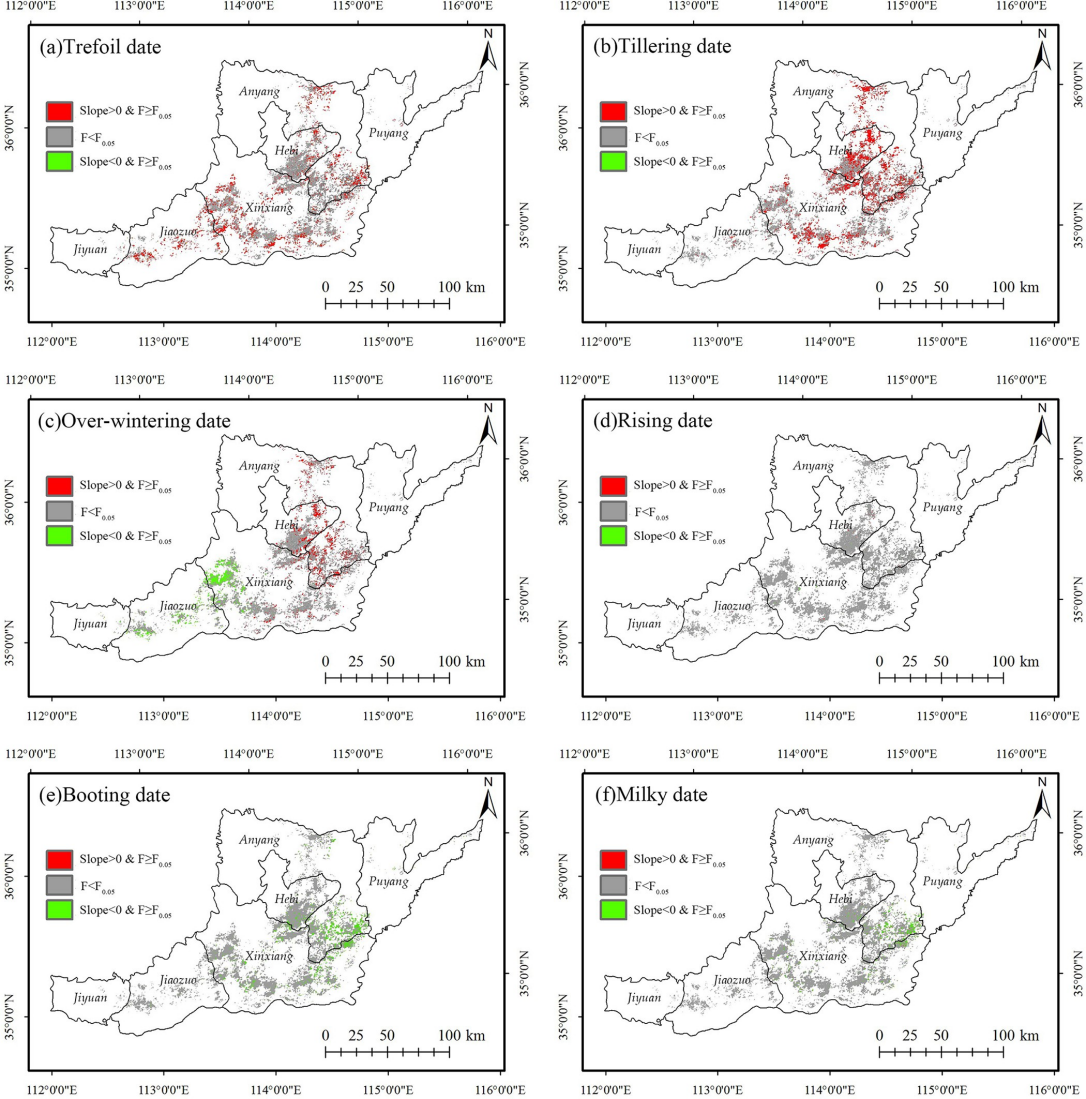
Significance of the changing trend of the winter wheat phenological dates in Northern Henan from 2003 to 2018. The administrative boundary data comes from Resource and Environment Science and Data Center (https://www.resdc.cn/).

Figs 5 and 6 show the slopes and significance of univariate regression for different phenological dates at the pixel scale in northern Henan. The average regression slopes and standard deviations for all winter wheat pixels in each phenological period are shown in Table 4.

**Table 4:** The mean linear regression slopes of the key phenological dates of the winter wheat in Northern Henan.

Index	Trefoil	Tillering	Overwintering	Rising	Booting	Milky
Regression slope (d/a)	0.6919	0.4363	0.0737	-0.0104	-0.3968	-0.2914
Standard deviation (d/a)	0.2487	0.2786	0.4258	0.2332	0.2086	0.2299

The “d/a” in the table represents the average number of days per year, where “d” stands for days and “a” for annus.

According to Figs 5 and 6, the changing trend of the winter wheat phenological dates before and after overwintering in northern Henan is significantly different. The average regression slope of overwintering, trefoil, and tillering dates is greater than 0, and the interannual change rate is 6.92 d/10a, 4.36 d/10a, and 0.74 d/10a, showing a delayed trend. The trefoil and tillering stages before winter were significantly delayed. Significant trend towards earlier wintering in the west and later in the east. The average regression slope of the rising, booting, and milky dates after winter is less than 0, and the interannual change rate is -0.1 d/10a, -3.97 d/10a and -2.91 d/10a, showing an advance trend. Among them, the advance trend of the rising stage is not significant, the booting and the milky stages are advanced overall, and some parts of the central and eastern regions are significantly advanced. From 2003 to 2018, the growth of winter wheat in northern Henan from the trefoil stage to the milky stage showed a trend of shortening, consistent with the relevant research results [32, 33].

#### 4.4.2 Differences in regional changes of the phenological stages in northern Henan

**Fig 7:**
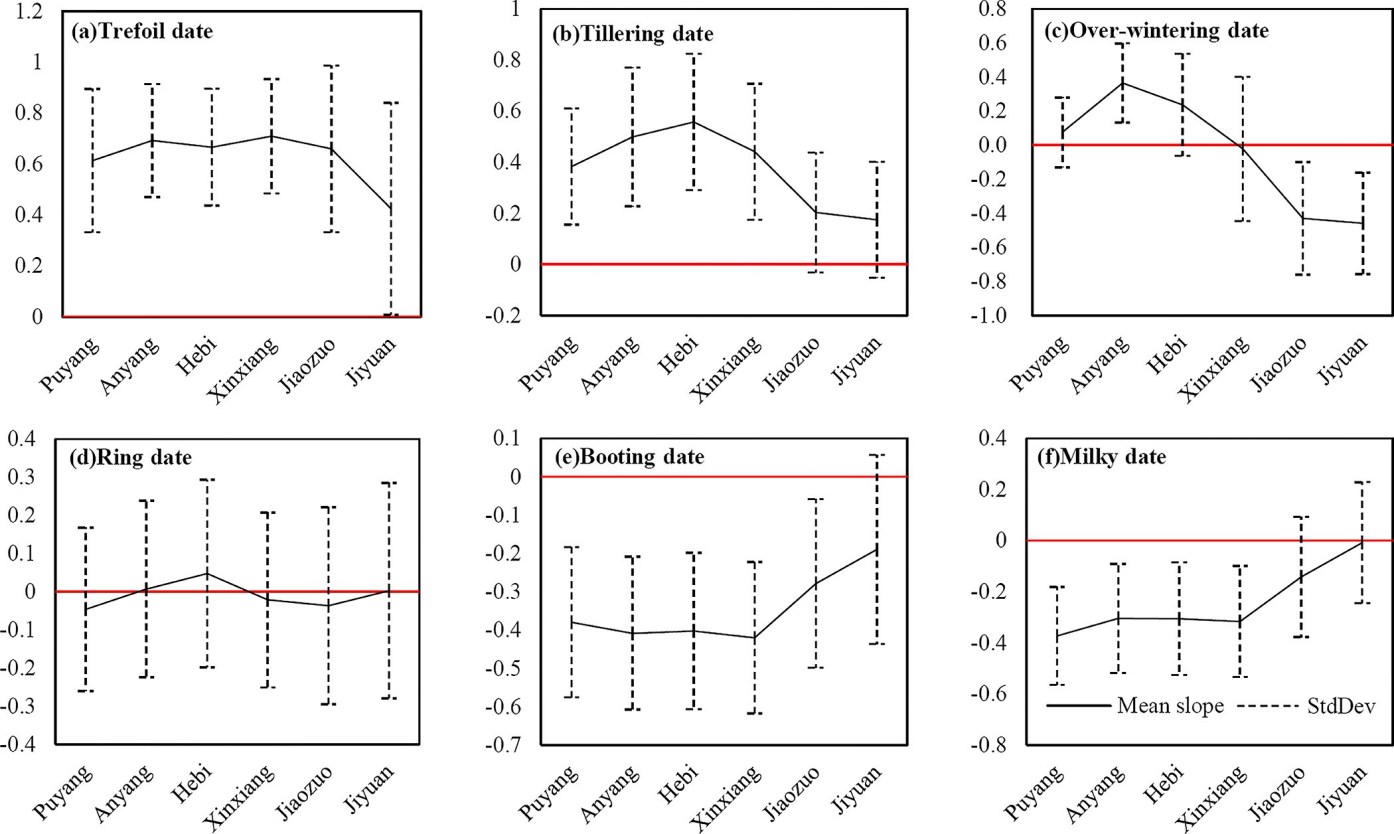
Zonal statistics of the slope of the linear regression equation for the key phenological dates of the winter wheat. Cities ordered from east to west.

The average regression slope value of each city in the northern Henan was calculated to evaluate the difference of changing trend of phenological periods in the cities (Fig 7).

The statistical results showed that the regression slopes of the trefoil and tillering dates in all cities in northern Henan were greater than 0, showing an overall delayed trend. The regression slope of Xinxiang was the largest. In the meantime, the delayed trend of the trefoil stage in Jiyuan and the delayed trend of the tillering stage in Jiyuan and Jiaozuo in the west were significantly lower than in other cities. The difference in the regression slope between the east and the west during the winter was obvious: Puyang, Anyang, and Hebi in the east were greater than 0; Xinxiang in the middle was -0.0219, close to 0; Jiaozuo and Jiyuan in the west were less than 0. The overwintering stage showed a changing pattern in the eastern cities delayed, and the western cities advanced; The northwest of Xinxiang was ahead of schedule, and the southeast was behind schedule. The internal difference was obvious in northern Henan (Fig 5), but the average value was close to 0. The absolute value of the regression slope of the rising date of each city was small, with an inconspicuous overall changing trend. The changing trend of the booting date and milky date in the 6 cities was consistent, and the regression slopes were less than 0, with an obvious advancing trend; the advancing rate of Jiaozuo and Jiyuan in the west was significantly lower than that of the cities in the middle and east.

#### 4.4.3 Response of the phenological period to climate change

The correlation between the monthly temperature and precipitation data of 27 meteorological stations in northern Henan and the extracted value of the key phenological period (DOY) of the winter wheat from 2003 to 2018 was analyzed to clarify the relationship between climate change and the phenological period. Due to the lag effect of temperature and precipitation on the growth period of the winter wheat, the temperature and precipitation data were taken from the data of the current month and the previous month (Table 5).

**Table 5:** Correlation analysis between the start time of key phenological stages and meteorological elements.

TR		TL		OW		RS		BT		ML	
(Oct. 16-Nov. 17)		(Nov. 17-Dec. 19)		(Dec. 19-Jan. 17)		(Feb. 10—Mar. 6)		(Apr. 7- May. 1)		(May. 9—Jun. 2)	
Index	*R*	Index	*R*	Index	*R*	Index	*R*	Index	*R*	Index	*R*
Oct *T*_*m*_	0.044	Nov *T*_*m*_	-.423**	Dec *T*_*m*_	0.03	Feb *T*_*m*_	-.197**	Mar *T*_*m*_	-.330**	Apr *T*_*m*_	-.400**
Oct *T*_*max*_	-0.06	Nov *T*_*max*_	-.460**	Dec *T*_*max*_	0.004	Feb *T*_*max*_	-.233**	Mar *T*_*max*_	-.299**	Apr *T*_*max*_	-.440**
Oct *T*_*min*_	.203**	Nov *T*_*min*_	-.218**	Dec *T*_*min*_	0.085	Feb *T*_*min*_	-.111*	Mar *T*_*min*_	-.276**	Apr *T*_*min*_	-.314**
Oct *P*	-0.005	Nov *P*	.174**	Dec *P*	-0.047	Feb *P*	.125**	Mar *P*	-.235**	Apr *P*	-.175**
Nov *T*_*m*_	.326**	Dec *T*_*m*_	.316**	Jan *T*_*m*_	.130**	Mar *T*_*m*_	-.287**	Apr *T*_*m*_	-.364**	May *T*_*m*_	-.361**
Nov *T*_*max*_	.201**	Dec*T*_*max*_	.244**	Jan *T*_*max*_	0.012	Mar *T*_*max*_	-.298**	Apr *T*_*max*_	-.396**	May *T*_*max*_	-.424**
Nov *T*_*min*_	.416**	Dec *T*_*min*_	.322**	Jan *T*_*min*_	.205**	Mar *T*_*min*_	-.202**	Apr *T*_*min*_	-.285**	May *T*_*min*_	-.171**
Nov *P*	.136**	Dec *P*	-0.078	Jan *P*	-.259**	Mar *P*	-.102*	Apr *P*	-.150**	May *P*	-.248**

*T*_*m*_, *T*_*max*_, *T*_*min*_ and *P* are monthly mean temperature, monthly maximum temperature, monthly minimum temperature and monthly cumulative precipitation respectively; *R* is Pearson correlation coefficient

** indicates significant correlation at 0.01 (two-tailed), and

* indicates significant correlation at 0.05 (two-tailed).

According to Table 5, the start time of the phenology of winter wheat in northern Henan is significantly correlated with the temperature. The trefoil date had a significant positive correlation with the minimum temperature (*T*_*min*_) in October, the mean temperature (*T*_*m*_), the maximum temperature (*T*_*max*_), and the *T*_*min*_ in November. In other words, the higher the temperature, the later the trefoil stage started, which is consistent with the research results in literature [34]. The overwintering date was positively correlated with the *T*_*m*_ and *T*_*min*_ in January. The higher the *T*_*m*_ and *T*_*min*_ in January, the later the overwintering stage was. The post-winter phenology, such as the rising, booting, and milky dates, had a significant negative correlation with the temperature of the corresponding month. In other words, the increasing temperature could advance the rising, booting, and milky stages of the winter wheat, as concluded in literature [15]. It is worth noting that the tillering date had a significant positive correlation with the temperature in November and a significant negative correlation with the temperature in December. Since the pre-winter NDVI peak was extracted as tillering date at the tillering stage, a higher temperature in November could promote the occurrence of tillering bringing the peak forward. If the higher temperature continues into December, the proportion of pre-winter tillers would continue to increase, and the peak would be delayed.

For precipitation (*P*), *P* of November was significantly and positively correlated with the trefoil dates and tillering dates. Next, the *P* of January was significantly negatively correlated with the overwintering dates. Then, the *P* of February was significantly positively correlated with the rising dates. And the *P* of March was significantly negatively correlated with the rising dates and booting dates. Finally, the *P* of April and May was significantly negatively correlated with the booting dates and milky dates.

The response of the start time in different phenological stages of winter wheat to the changes in meteorological elements was different. In general, the increase in temperature was the main factor influencing the delay of the start time of the pre-winter stages, the advance of the start time of the post-winter stages and the shortening of growth period of winter wheat [16].

## 5. Discussions and conclusions

### 5.1 Discussions

#### 5.1.1 Factors affecting the accuracy of tillering date extraction

Generally speaking, the tillering wheat seedlings in the field can reach 70% at the pre-winter peak time of NDVI in northern Henan. That is, the maximum NDVI value of pre-winter appeared after the observed tillering date of the agro-meteorological station (The date when the tillering wheat seedlings in the field reach 50%). However, the degree of tillering pre-winter varied between regions. For example, the MAE between the observation date of the tillering stage and the extracted date of the pre-winter NDVI peak at the Xinxiang agro-meteorological station was about 21 d, and at the Tangyin agro-meteorological station was 33d in 2018. Meanwhile, there can also be significant differences between different years in the same region. Due to the effect of low temperatures before winter, 50% of the tillers in the whole field could occur after the overwintering stage. For example, the date of tillering stage observed at the Puyang agro-meteorological station in 2012 was March 4th, significantly different from the average observed value over the years. These uncertainties led to the low accuracy of remote sensing extraction of the tillering date, which is the main reason why the tillering stage is rarely mentioned in remote sensing monitoring studies of winter wheat phenology [34].

The study found that, the MAE between the extracted pre-winter maximum NDVI dates and the observed tillering dates at the 9 agro-meteorological stations in northern Henan in 2018 was 17 d. It is worth noting that the extracted dates were all later than the observed dates except for the Qinyang station. Subtracting 17d from the extracted dates resulted in a decrease in MAE and RMSE of the tillering dates decreased to 7.6 d and 9.2 d (Table 6), and the total MAE and RMSE decreased to 7.0 d and 9.7 d. Similarly, on Xinxiang station from 2003 to 2018, the MAE and RMSE of the tillering dates decreased to 7.3 d and 8.7 d, and the total MAE and RMSE decreased to 7.4 d and 9.6 d. Thus, the tillering date can be calculated by subtracting the MAE from the extracted pre-winter maximum NDVI dates, which will increase the accuracy.

**Table 6:** Error statistics for tillering dates extraction when subtracting 17 days from the extracted dates.

Phenological stage	Phenological date	PY	HX	LZ	TY	HB	XX	FQ	JY	QY	MAE (d)	RMSE (d)
Tillering	Observed	328	318	314	318	312	322	322	334	338	7.6	9.2
	Extracted -17	328	328	312	328	320	320	328	320	320		

#### 5.1.2 Is the post-winter maximum NDVI date of the winter wheat the heading date or booting date?

**Fig 8:**
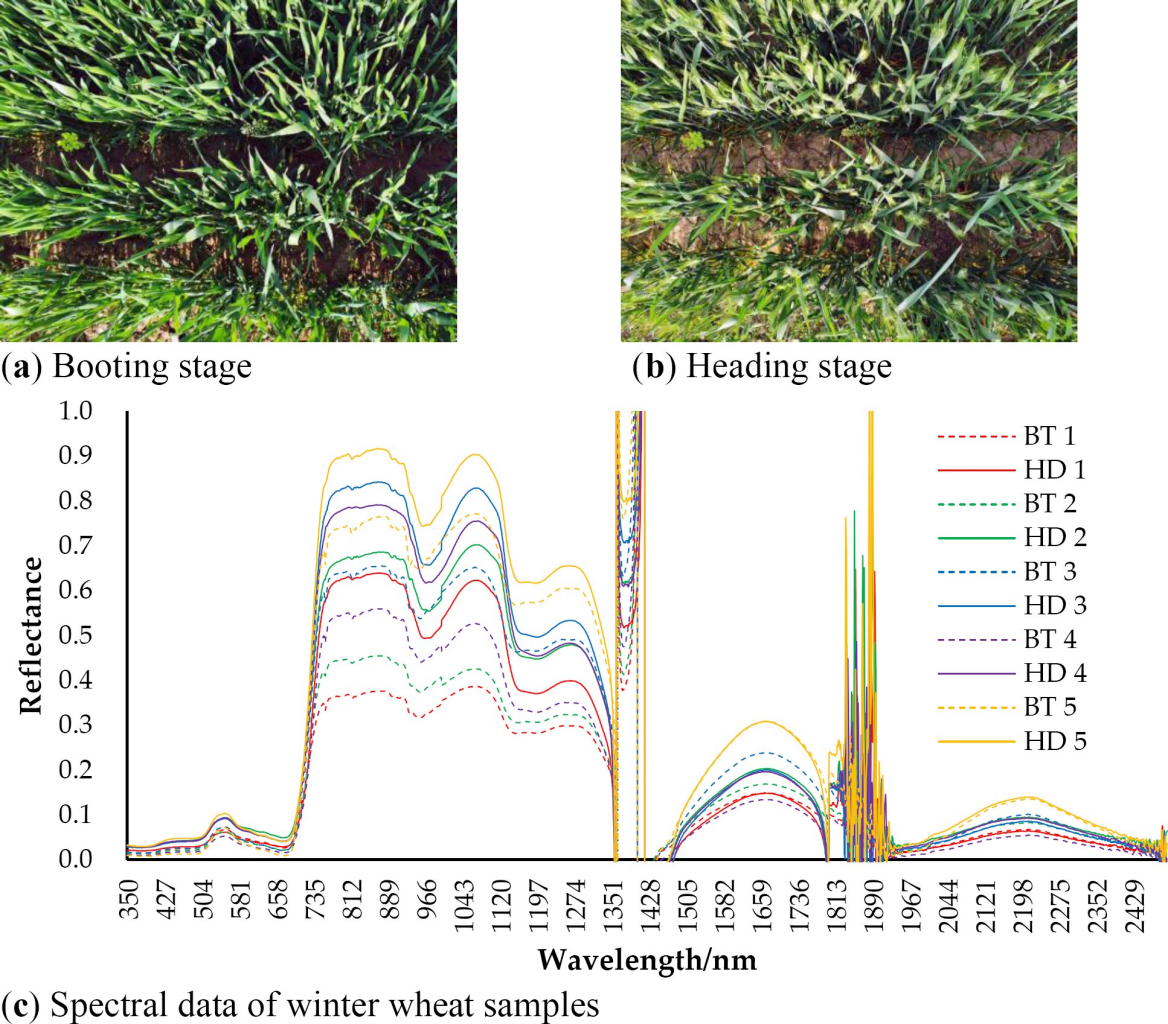
Winter wheat samples and their spectral data at booting and heading stage: (a) Winter wheat at booting stage; (b) Winter wheat at heading stage; (c) Spectral data of winter wheat samples at booting and heading stage. HD for heading stage, BT for booting stage.

Many studies defined the date of the post-winter maximum NDVI of the winter wheat as the heading date, but found that the extracted dates were generally earlier than the dates recorded by the agro-meteorological stations [15, 20, 24, 35]. However, some studies found that, the leaf area index and NDVI peaked at the booting stage rather than the heading stage [29–31]. To determine the corresponding phenological stage of the peak NDVI of winter wheat, spectroscopic measurements were performed on winter wheat samples from the two periods (Fig 8) using ASD portable spectroscopy (FieldSpec 4 Wide-Res, 350-2500nm). The mean NDVI values were 0.9109 and 0.8885 for the winter wheat samples at the booting and heading stages, respectively. It was clear that the peak of winter wheat NDVI occurs at the booting stage in the north of Henan Province. The lack of strict distinction between the booting and the heading stages may be one of the reasons why the extracted heading dates were earlier than the observed dates in some studies [29–31].

#### 5.1.3 Related factors influencing the spatio-temporal variation of phenological dates of winter wheat in northern Henan Province

Many studies have shown clear latitudinal zoning in the winter wheat phenological period [16, 17, 20]. However, this study found no such zoning in the spatio-temporal characteristics of winter wheat phenological dates in northern Henan. Furthermore, the spatio-temporal variations of several phenological dates, including the tillering date and the overwintering date, exhibited anisotropic longitudinal zonation characteristics (Figs 4–7).

The Taihang Mountains, located in the northwestern part of northern Henan, may contribute to the variation in temperature and rainfall between the western and eastern regions of northern Henan. This results in a longitudinal zonality of the spatio-temporal changes of the phenological period. Furthermore, local farmers adjust their farming practices to cope with the effects of climate change, ensuring optimal crop growth despite the changing climate. For example, if the temperature is high in October, farmers delay the sowing time [32]. Generally, climate change, adjustments in ploughing techniques, and topographical factors result in spatio-temporal changes and characteristics of winter wheat phenology in the study region.

#### 5.1.4 Limitations and uncertainties

Phenological date extraction accuracy can be impacted by numerous factors. The choice of filtering and reconstruction methods can impact the extraction results of the phenological dates. For instance, HANTS filtering has limitations on asymmetric data. If the VI peak is biased to the left or right, the extraction value would be delayed or advanced accordingly. Additionally, researchers had different ideas about the phenological dates corresponding to the turning points of the winter wheat NDVI curve. As mentioned earlier, there are different views on the phenological period corresponding to the maximum NDVI value after winter [20, 24, 29–31]. Another example, the date of the pre-winter maximum NDVI, literature [17] defined it as the tillering date, while literature [36] defined it as the beginning of the overwintering stage. Moreover, the observations of the agro-meteorological stations are strongly influenced by the subjective influence of the observers. For example, the observation standard of the China agro-meteorological station for the start of the overwintering period is “the plants basically stops growing and the tillers no longer increase or grow slowly (this can be based on the last day when the 1st 5-day mean temperature drops to 0°C)” [28]. The observed values may be related to the growth status of winter wheat or to the mean daily temperature. Therefore, the uncertainty of the observed values might be one of the reasons for the significant validation errors of the extracted overwintering dates of the winter wheat in northern Henan Province.

The segment turning point method used in this study could easily extract the turning points from the winter wheat NDVI curve. However, the extraction accuracy was limited by the fact that the NDVI curves were not interpolated to 1 d. Furthermore, the corresponding relationship between the turning points of the winter wheat NDVI curves and the ground observations of phenological dates in different regions might vary. This study has not yet compared the effects of this method in other regions. To address these issues, future studies can use curve-fitting equations that interpolate NDVI time intervals to 1d to improve extraction accuracy. Meanwhile, future studies can compare of the effects of different filters and curve-fitting methods on extraction accuracy, as well as compare of the extraction accuracy in different regions, to further optimize the method and verify its effectiveness and adaptability.

### 5.2 Conclusion

In this paper, the key phenological dates of winter wheat in northern Henan from 2003 to 2018 were extracted using MODIS NDVI time series data and the segment turning point method. On this basis, the spatio-temporal variation characteristics of key phenological dates and the effects of temperature and precipitation on the phenological changes were analyzed.

The results showed that, the spatial differences of each phenological date in northern Henan were pronounced in 2018. The trefoil stage, tillering stage, overwintering stage, and rising stage were roughly bounded by 114°E, and the western region was earlier than the eastern region. From 2003 to 2018, the interannual variation rates of the start dates of the trefoil stage, tillering stage, overwintering stage, rising stage, booting stage, and milky stage of the winter wheat in northern Henan were 6.92 d/10a, 4.36 d/10a, 0.74 d/10a, -0.1 d/10a, -3.97 d/10a, and -2.91 d/10a. The trefoil and tillering stages were significantly delayed, and the overwintering stage was advanced in the west and delayed in the east. At the same time, the rising stage, booting stage, and milky stage of post-winter showed an overall advance trend, of which the rising stage was advanced but not significantly, and the booting and milky stages were significantly advanced in some parts of the central and eastern regions. The correlation analysis showed that the increase in temperature was the main factor influencing the delay of the pre-winter phenological stages and the advance of the post-winter phenological stages.

The research results could provide a reference for remote sensing monitoring of winter wheat phenology and research on the response of phenological stages to climate change.
